# Elevated Systemic and Parasite—Antigen Stimulated Levels of Type III IFNs in a Chronic Helminth Infection and Reversal Following Anthelmintic Treatment

**DOI:** 10.3389/fimmu.2018.02353

**Published:** 2018-10-23

**Authors:** Anuradha Rajamanickam, Saravanan Munisankar, Yukthi Bhootra, Chandrakumar Dolla, Thomas B. Nutman, Subash Babu

**Affiliations:** ^1^National Institute of Health-NIRT-International Center for Excellence in Research, Chennai, India; ^2^National Institute for Research in Tuberculosis, Chennai, India; ^3^Laboratory of Parasitic Diseases, National Institute of Allergy and Infectious Diseases, National Institutes of Health, Bethesda, MD, United States

**Keywords:** Type III IFNs, DC subsets, IFN lambda-1, IFN lambda-2, IFN lambda-3, *Strongyloides stercoralis*, helminths

## Abstract

Type III IFNs are important players in immunity to viral and bacterial infections. However, their association with helminth infections has not been examined. To explore the association of Type III IFNs with *Strongyloides stercoralis* (*Ss*) infection, we examined the systemic levels of IFN lambda-1, IFN lambda-2 and IFN lambda-3, IL-10, and CXCL10/IP-10 in *Ss* infected (INF, *n* = 44), helminth—uninfected (UN, *n* = 44) and in post-treatment INF individuals. We also examined the levels of IFN lambda-1, IFN lambda-2 and IFN lambda-3, IL-10, and CXCL10/IP-10 in whole blood culture supernatants stimulated with *Ss* somatic antigens, or PPD or LPS. Finally, we performed correlations of systemic Type III IFN levels with absolute numbers of dendritic cell subsets. *Ss* infection is characterized by elevated systemic levels of IFN lambda-1, IFN lambda-2 and IFN lambda-3, IL-10, and CXCL10/IP-10 in comparison to UN individuals and a significant reduction following anthelmintic treatment. *Ss* infection is also characterized by elevated levels of unstimulated or *Ss* antigen stimulated levels of IFN lambda-1, IFN lambda-2 and IFN lambda-3, CXCL10/IP-10 and a significant reduction following treatment. In addition, *Ss* infection is characterized by increased numbers of plasmacytoid and myeloid dendritic cells in comparison to UN individuals, with a significant reduction following anthelmintic treatment of INF individuals. Finally, *Ss* infection exhibits a significant positive correlation between the systemic levels of IFN lambda-2 and IFN lambda-3 and the numbers of plasmacytoid dendritic cells. Thus, *Ss* infection is characterized by elevations in systemic and antigen—induced levels of Type III IFNs, which is positively associated with the numbers of plasmacytoid dendritic cells and reversed upon anthelmintic treatment.

## Introduction

Type III infterferons (IFNs), also termed IFN-λ, are part of a cytokine family that shares functional similarity with Type I IFNs (1, 2). The members of the Type III IFNs in humans are IFN lambda-1, IFN lambda-2, IFN lambda-3 along with a poorly characterized IFN-λ4 (3, 4). Type III IFNs are typically produced by epithelial cells in response to viral infection, upon sensing of pattern recognition receptors, including members of the RIG-I-like receptors or Toll-like receptors (5, 6). Myeloid lineage cells are also now known to be important producers of Type III IFNs, with plasmacytoid dendritic cells being the major producers (7–9). Type III IFNs have typically been associated with protection against viral infections, including hepatitis B virus, hepatitis C virus, human Cytomegalovirus, and Herpes Simplex Virus-1 (1, 2). They are thought to contribute to specialized immune mechanisms that protect epithelial surfaces against pathogenic microbes. More recent evidence suggests that Type III IFNs also play a role in host anti-bacterial and anti-fungal defense mechanisms, including against *Salmonella, Listeria, and Aspergillus* infections (10–12). However, their role in helminth infections has not, to our knowledge, been explored.

*Strongyloides stercoralis* (*Ss*), a soil transmitted nematode that resides in the small intestine of humans, infects approximately 30–100 million people worldwide (13). *Ss* infection is typically associated with increased production of Type 2 and regulatory cytokines and decreased production of Type 1 and Type 17 cytokines (14, 15). The association of Type 1 and Type 3 IFNs with *Ss* infection is not known. *Ss* infection serves as good model to study its association of Type III IFN as the parasite primarily resides within the lumen of the intestine where it very likely interacts with the GI tract's mucosal surface and where it could induce IFN responses (16). To examine the role played by Type III IFN in *Ss* (and presumably other soil transmitted helminth) infection, we measured both the expression systemically of these Type III IFNs and also their induction in whole blood by parasite antigen before and following definitive therapy for *Ss*. Because dendritic cells can be a source of these Type III interferons (8, 9), we tried to understand the relationship between the Type III IFNs and circulating dendritic cell subsets.

## Materials and methods

### Ethics statement

All individuals (age between 18–65 years) were examined as part of a natural history study protocol approved by Institutional Review Boards of the National Institute of Allergy and Infectious Diseases (USA) and the National Institute for Research in Tuberculosis (India), and informed written consent was obtained from all participants.

### Study population

We studied 88 individuals comprised of 44 clinically asymptomatic, *Ss*-infected (hereafter INF) individuals and 44 *Ss*-uninfected, endemic healthy (hereafter UN) individuals in Tamil Nadu, South India (Table 1). These individuals were all recruited from a rural population by screening of individuals for helminth infection by stool microscopy and serology as described previously (17–19). Hookworm and Ascaris infection was ruled out by Fecal Egg count (FEC) and PCR methods. None had previous anthelmintic treatment or a history of prior helminth infection. Follow up among the INF individuals was performed at 6 months following treatment.

**Table 1:** Baseline demographics of study population.

**Study demographics**	**INF**	**UN**
Number of subjects recruited	*n* = 44	*n* = 44
Gender (Male/Female)	24/20	23/21
Median age (range)	38 (20–60)	40 (20–60)
NIE ELISA	Positive	Negative

*Ss* infection was diagnosed by the presence of IgG antibodies to the recombinant NIE antigen, a 31-kDa antigen derived from *S*. *stercoralis* L3 parasites as described previously (18, 19). Filarial infection was excluded in all study participants by virtue of being negative in tests for circulating filarial antigen. All INF individuals were treated with single dose of ivermectin (12 mg) and albendazole (400 mg), and follow—up blood draws were obtained 6 months later.

We also studied a group of 44 individuals with hookworm infection, who were positive by stool microscopy and PCR (hereafter INF). Stool samples were collected, transported to the laboratory at ambient temperatures, and examined by direct microscopy and by formal-gasoline concentration techniques, as described previously (17). Stool microscopy was used to exclude the presence of other intestinal helminths including Ascaris, Strongyloides, Trichuris, Enterobius, Taenia, and Hymenolepsis.

### Parasite and control ag

Saline extracts of *S. stercoralis* somatic larval Antigen (*Ss* Ag) was used as parasite antigenic stimuli, PPD was used as non-parasite antigen control and LPS was used as positive control. The crude Ss larval antigen was extracted with distilled water accompanied by alumina in ice and then subjected to five periods of sonication, each of 1 min. The supernatant was collected by centrifugation at 12,000 g at 4°C for 30 min. The protein content was determined by the method of Bradford.

Excretory–secretory (ES) antigen from hookworm larvae was used as antigenic stimuli for hookworm infected individuals. Hookworm samples also stimulated with P/I as a control antigens. Final concentrations were 10 μg/ml for *Ss* Ag, PPD, ES Ag, and LPS. Phorbol ester (PMA) and ionomycin at concentrations of 12.5 and 125 ng ml, respectively, were used as the positive control stimuli.

### *In vitro* culture

Whole blood cell cultures were performed to determine the *in vitro* responses to antigens in a subset of INF (*n* = 22) and UN (*n* = 22) individuals and in INF individuals 6 months following treatment. Briefly, whole blood was diluted 1:1 with RPMI1640 medium supplemented with penicillin/streptomycin (100 U/100 mg/ml), L-glutamine (2 mM), and HEPES (10 mM; all from Invitrogen) and distributed in 12-well tissue culture plates (Costar). The cultures were then stimulated with *Ss* Ag, PPD, or LPS or with medium alone. The hookworm infected samples were stimulated with ES Ag or P/I or with with medium alone at 37°C for 18 h. After 18 h, the supernatants were collected and stored at −80°C until further use.

### Measurement of type III IFNs

Plasma and whole blood culture supernatant levels of IFN lambda-1(IL-29), IFN lambda-2 (IL-28A), IFN lambda-3(IL-28B), IL-10, and CXCL10/IP-10 were measured using ELISA kits (R&D Systems, Minneapolis, MN, USA) according to the manufacturer's instructions. The detection limits were as follows: IFN lambda-1, 32.5 pg/ml; IFN lambda-2, 25 pg/ml; IFN lambda-3, 15.6 pg/ml; IL-10, 31.2 pg/ml; CXCL10/IP-10, 62.5 pg/ml.

### *Ex vivo* analysis

All antibodies used in the study were from BD Biosciences (San Jose, CA), BD Pharmingen (San Diego, CA), eBioscience (San Diego, CA), or R&D Systems (Minneapolis, MN). Whole blood was used for *ex vivo* phenotyping and it was performed on all 88 individuals. Briefly, 250 μl aliquots of whole blood were added to a cocktail of monoclonal antibodies specific for dendritic cells (DC) subsets. Phenotyping of DC was performed using antibodies directed against HLA- DR, lineage cocktail (CD3, CD14, CD16, CD19, CD20, CD56) FITC (clone SJ25C1, SK7, MΦp9, L27, NCAM16.2, 3G8 BD) CD123-PE (clone 9F5; BD) CD11c- APC (clone S-HCL-3; BD). Plasmacytoid DC (pDC) were classified as Lin– HLA-DR^+^ CD123^+^ and myeloid DC (mDC) as Lin– HLA-DR^+^ CD11c^+^. Following 30 min of incubation at room temperature erythrocytes were lysed using 2 ml of FACS lysing solution (BD Biosciences Pharmingen), cells were washed twice with 2 ml of PBS and suspended in 200 μl of PBS (Lonza, Walkersville, MD). Eight- color flow cytometry was performed on a FACS Canto II flow cytometer with FACSDIVA software, version 6 (Becton Dickinson). The gating was set by forward and side scatter, and 100 000 gated events were acquired. Data were collected and analyzed using FLOW JO software (TreeStar, Ashland, OR). Leukocytes were gated using CD45 expression compared to side scatter. Absolute counts of the subpopulations were calculated based on the equation: Absolute number/mm^3^ of DC subset = [percent of subset × total number of white blood cells per mm^3^]/100). The gating strategy is shown in Supplementary Figure 1.

### Statistical analysis

Data analyses were performed using GraphPad PRISM 7 (GraphPad Software, Inc., San Diego, CA, USA). Geometric means (GM) were used for measurements of central tendency. Statistically significant differences were analyzed using the non-parametric Mann-Whitney *U*-test and Wilcoxon matched pair test. Multiple comparisons were corrected using the Holm's correction. Correlations were calculated by the Spearman rank correlation test.

## Results

### Study population characteristics

The baseline characteristics and demographics of the study population are shown in Table 1. No significant differences in age, gender, socioeconomic status, or geographical location were observed between the two groups.

### *Ss* infection is associated with increased systemic levels of type III IFNs and reversal following treatment

**Figure 1:**
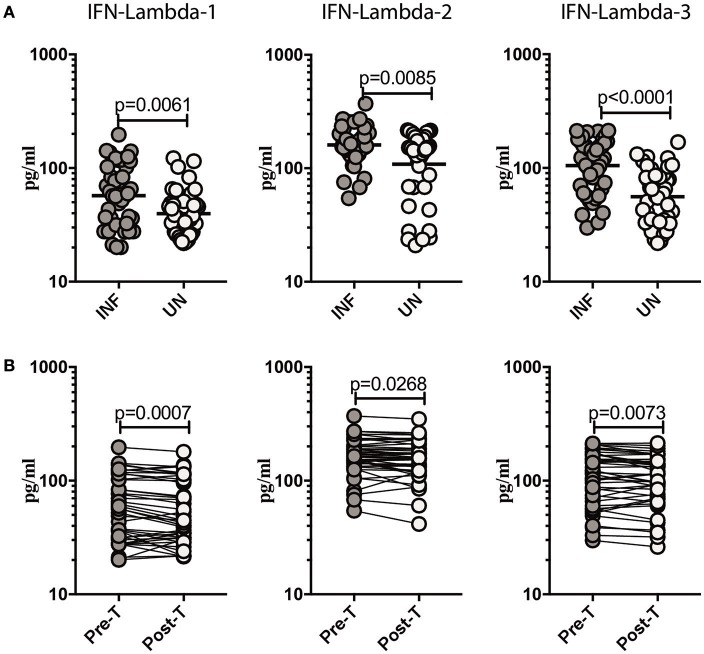
*Ss* infection is associated with elevated plasma levels of Type III IFNs and reversal following treatment. **(A)** The plasma levels of Type III IFNs, IFN-lambda-1, IFN-lambda-2, and IFN-lambda-3 were measured in *Ss*-infected [INF] (*n* = 44) or un-infected [UN] (*n* = 44) individuals. The data are represented as scatter plots with each circle representing a single individual. *P*-values were calculated using the Mann–Whitney *U*-test with Holms correction for multiple comparisons. **(B)** The plasma levels of Type III IFNs, IFN-lambda-1, IFN-lambda-2, and IFN-lambda-3 were measured in *Ss*-infected INF individuals at pre-treatment [pre-T] (*n* = 44) and 6 months following treatment post-treatment [post-T] time points. The data are represented as line graphs with each line representing a single individual. *P*-values were calculated using the Wilcoxon signed rank test.

To determine if *Ss* infection altered the systemic levels of Type III IFNs, we measured the circulating plasma levels of IFN lambda-1, IFN lambda-2, and IFN lambda-3 in INF and UN individuals. As shown in Figure 1A, INF had significantly higher plasma levels of IFN lambda-1, (GM of 57.16 pg/ml in INF compared to 39.7 pg/ml in UN, *p* = 0.0061); IFN lambda-2, (GM of 160.8 pg/ml in INF compared to 108.6 pg/ml in UN, *p* = 0.0085) and IFN lambda-3, (GM of 105.1 pg/ml in INF compared to 55.93 pg/ml in UN, *p* < 0.0001) in comparison to UN individuals. To determine the effect of treatment on Type III IFN levels, we measured these parameters in INF individuals 6 months following anthelmintic treatment (post-T) and compared them to levels before treatment (pre-T). As shown in Figure 1B, INF individuals had significantly lower plasma levels of IFN lambda-1 (GM fold change of 1.18 compared to pre-T vs. post-T, *p* = 0.0007), IFN lambda-2 (GM fold change of 1.14 compared to pre-T vs. post-T, *p* = 0.0268) and IFN lambda-3 (GM fold change of 1.15 compared to pre-T vs. post-T, *p* = 0.0073) at post-treatment in comparison to pre-treatment levels. We also wanted to determine if these altered levels of Type III IFNs are specific to *Ss* infection or common to other intestinal helminth infections. We therefore, measured IFN lambda-1, IFN lambda-2 and IFN lambda-3 in hookworm infected and UN individuals. However, we did not observe any significant difference in the levels of Type III IFNs between the 2 groups (Supplementary Figure 1). Thus, *Ss* infection (but not hookworm infection) is associated with elevated plasma levels of Type III IFNs and reversal following treatment.

### *Ss* infection is associated with increased parasite antigen stimulated levels of type III IFNs

**Figure 2:**
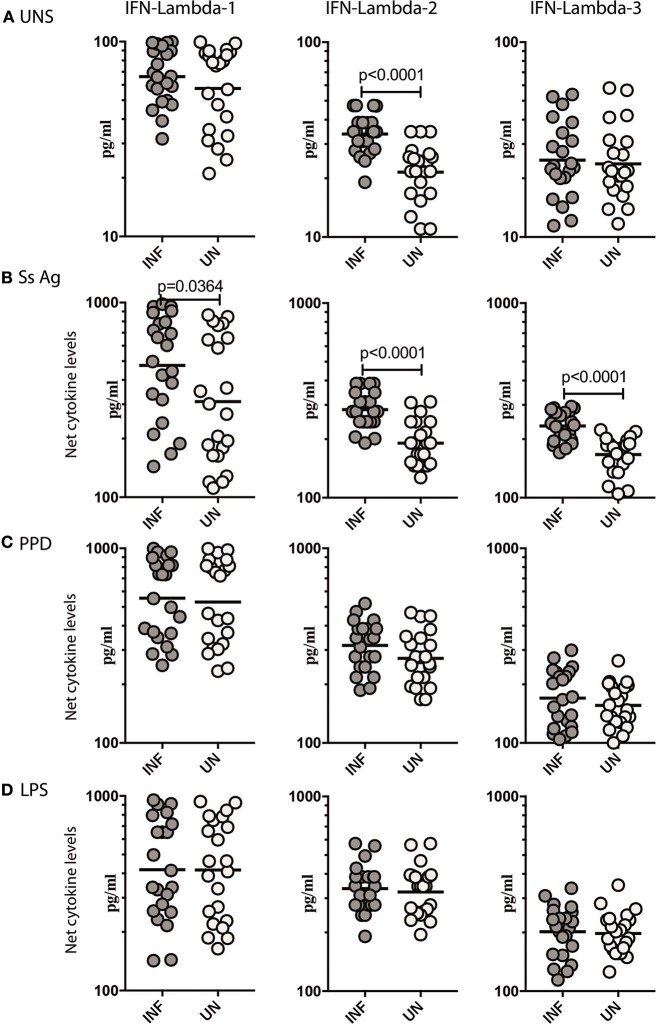
*Ss* infection is associated with elevated unstimulated and parasite antigen stimulated levels of Type III IFNs in whole blood supernatants. **(A)** The baseline or unstimulated (UNS) levels of Type III IFNs, IFN-lambda-1, IFN-lambda-2, and IFN-lambda-3 were measured in whole blood culture of *Ss*-infected [INF] (*n* = 22) or un-infected [UN] (*n* = 22) individuals. **(B)** The parasite antigen (*Ss* antigen) stimulated levels of Type III IFNs, IFN-lambda-1, IFN-lambda-2, and IFN-lambda-3 were measured in *Ss*-infected [INF] (*n* = 22) or un-infected [UN] (*n* = 22) individuals. **(C)** The PPD stimulated levels of Type III IFNs, IFN-lambda-1, IFN-lambda-2, and IFN-lambda-3. **(D)** The LPS stimulated levels of Type III IFNs, IFN-lambda-1, IFN-lambda-2, and IFN-lambda-3 were measured in *Ss*-infected [INF] (*n* = 22) or un-infected [UN] (*n* = 22) individuals. Net cytokine levels are calculated by subtracting the antigen stimulated values from unstimulated values. The data are represented as scatter plots with each circle representing a single individual. *P*-values were calculated using the Mann–Whitney *U*-test with Holms correction for multiple comparisons.

To determine what role, if any, parasite antigen plays in stimulating the production of Type III IFNs in *Ss* infection, we stimulated whole blood from INF and UN individuals with or without *Ss* antigen or LPS and measured the levels of IFN lambda-1, IFN lambda-2, and IFN lambda-3. As shown in Figure 2A, INF had significantly higher spontaneous production of IFN lambda-2 (GM of 33.78 pg/ml in INF compared to 21.54 pg/ml in UN, *p* < 0.0001) but not IFN lambda-3 or IFN lambda-1 in comparison to UN individuals. When stimulated with parasite antigen, the INF had significantly higher levels of IFN lambda-1 (GM of 475.4 pg/ml in INF compared to 310.1 pg/ml in UN, *p* = 0.0364) IFN lambda-2 (GM of 283.9 pg/ml in INF compared to 190.8 pg/ml in UN, *p* < 0.0001) and IFN lambda-3 (GM of 233.6 pg/ml in INF compared to 166.6 pg/ml in UN, *p* < 0.0001; Figure 2B). In contrast, as shown in Figure 2C, there were no differences in the levels induced by PPD and LPS in the 2 groups. Next, we determined whether these alterations were specific to *Ss* infection or induced by other helminth infections. We measured IFN lambda-1, IFN lambda-2 and IFN lambda-3 in whole blood of hookworm infected and UN individuals stimulated with ES Ag or PPD or P/I or media alone. There were no significant differences in the levels of Type III IFNs induced by ES Ag or PPD and P/I or at baseline between the 2 groups (Supplementary Figure 2). Thus, *Ss* infection (but not hookworm infection) was associated with increased levels of Type III IFNs when stimulated with parasite antigen.

### Anthelmintic treatment significantly decreases unstimulated, and parasite antigen stimulated levels of type III IFNs in *Ss* infection

**Figure 3:**
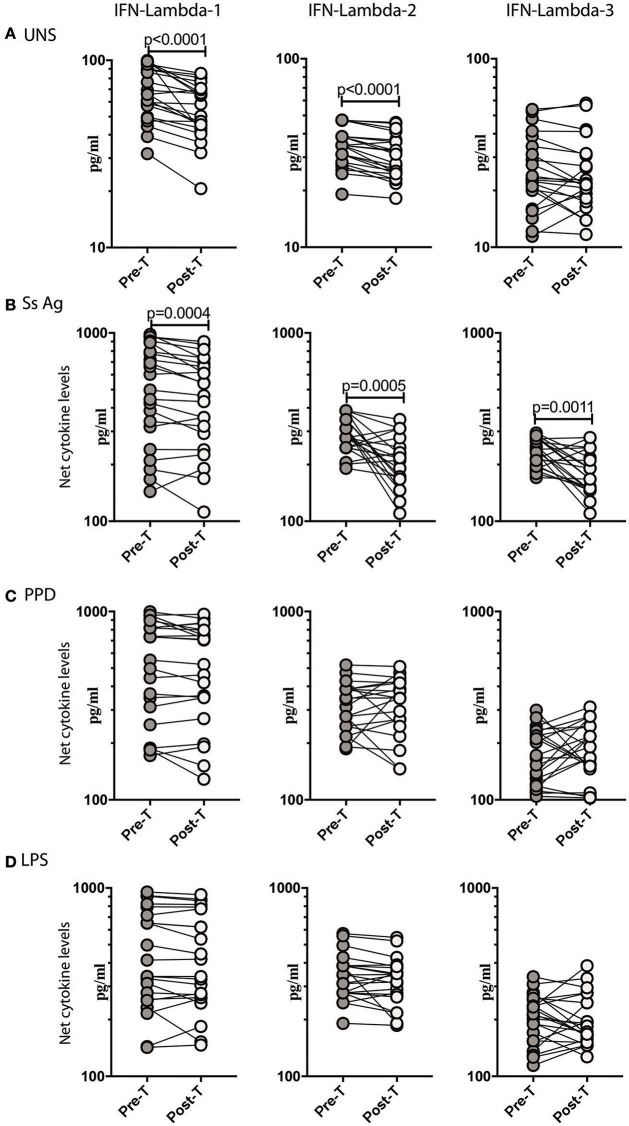
Anthelmintic treatment decreases unstimulated and parasite antigen stimulated levels of Type III IFNs in whole blood supernatants. **(A)** The baseline or unstimulated (UNS) levels of Type III IFNs, I IFN-lambda-1, IFN-lambda-2, and IFN-lambda-3 were measured in whole blood culture of INF (*n* = 22) individuals before (pre-T) and 6 months after anthelmintic treatment (post-T). **(B)** The parasite antigen (*Ss* antigen) stimulated levels of Type III IFNs, IFN-lambda-1, IFN-lambda-2, and IFN-lambda-3 were measured in INF (*n* = 22) individuals before (pre-T) and 6 months after anthelmintic treatment (post-T). **(C)** The PPD stimulated levels of Type III IFNs, IFN-lambda-1, IFN-lambda-2, and IFN-lambda-3 were measured in INF (*n* = 22) individuals before (pre-T) and 6 months after anthelmintic treatment (post-T). **(D)** The LPS stimulated levels of Type III IFNs, IFN-lambda-1, IFN-lambda-2, and IFN-lambda-3 were measured in INF (*n* = 22) individuals before (pre-T) and 6 months after anthelmintic treatment (post-T). Net cytokine levels are calculated by subtracting the antigen stimulated values from unstimulated values. Data are shown line diagrams with each line representing a single individual. *P*-values were calculated using the Wilcoxon matched pair test with Holms correction for multiple comparisons.

To determine if the increased production of Type III IFNs in *Ss* infection could be reversed by anthelmintic treatment, we stimulated whole blood from INF individuals with *Ss* antigen or PPD or LPS or media alone (unstimulated) before (pre-T) and 6 months after (post-T) treatment and measured the levels of IFN lambda-1, IFN lambda-2, and IFN lambda-3. As shown in Figure 3A, INF individuals had significantly lower unstimulated levels of IFN lambda-1 (GM fold change of 1.24, *p* < 0.0001) and IFN lambda-2 (GM fold change of 1.13 compared to pre-T vs. post-T, *p* < 0.0001) at post-treatment in comparison to pre-treatment levels. Similarly, INF individuals had significantly lower *Ss* antigen stimulated levels of IFN lambda-1 (GM fold change of 1.21, *p* = 0.0004) and IFN lambda-2 (GM fold change of 1.56, *p* = 0.0005), IFN lambda-3 (GM fold change of 1.29, *p* = 0.0011) at post-treatment in comparison to pre-treatment levels (Figure 3B). In contrast, INF individuals did not exhibit any significant difference in the PPD or LPS stimulated levels of IFN lambda-1, IFN lambda-2, and IFN lambda-3 post-treatment compared to pre-treatment levels (Figure 3C). Thus, anthelmintic treatment reversed the induction of Type III IFNs in *Ss* infection.

### *Ss* infection is associated with increased systemic and parasite antigen stimulated levels of IL-10, CXCL10/IP-10, and reversal following treatment

**Figure 4:**
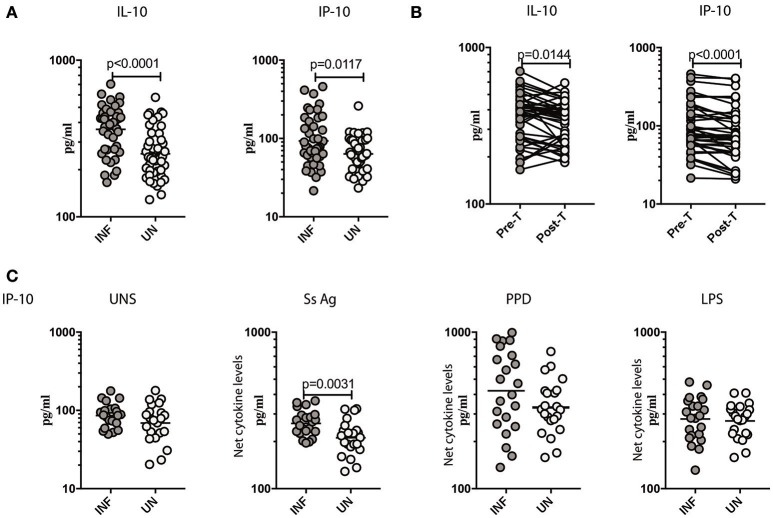
*Ss* infection is associated with increased systemic levels of IL-10, CXCL10/IP-10 and increased parasite antigeni stimulated levels of CXCL10/IP-10. **(A)** The plasma levels of IL-10 and CXCL10/IP-10 were measured in *Ss*-infected [INF] (*n* = 44) or un-infected [UN] (*n* = 44) individuals. The data are represented as scatter plots with each circle representing a single individual. *P*-values were calculated using the Mann–Whitney *U*-test with Holms correction for multiple comparisons. **(B)** The plasma levels of IL-10 and CXCL10/IP-10 were measured in *Ss*-infected INF individuals at pre-treatment [pre-T] (*n* = 44) and 6 months following treatment post-treatment [post-T] time points. The data are represented as line graphs with each line representing a single individual. *P*-values were calculated using the Wilcoxon matched pair test with Holms correction for multiple comparisons. **(C)** The baseline or unstimulated (UNS), parasite antigen (*Ss* antigen), PPD, and LPS stimulated levels of IL-10 and CXCL10/IP-10 were measured in whole blood culture supernants of *Ss*-infected [INF] (*n* = 22) or un-infected [UN] (*n* = 22) individuals. Net cytokine levels are calculated by subtracting the antigen stimulated values from unstimulated values. Data are shown line diagrams with each line representing a single individual. *P*-values were calculated using the Mann–Whitney *U*-test with Holms correction for multiple comparisons.

To measure the cellular transcriptional response to IFN stimulation, the levels of IL-10 (20) and CXCL10/IP-10 (21) was measured in *Ss* infected individuals before and after antihelmintic treatment. As shown in Figure 4A, INF individuals had significantly elevated levels of IL-10 (GM of 361.8 pg/ml in INF compared to 252.7 pg/ml in UN, *p* < 0.0001) and CXCL10/IP-10 (GM of 93.01 pg/ml in INF compared to 63.54 pg/ml in UN, *p* = 0.0117) in comparison to UN individuals. To determine the effect of treatment on the levels of regulatory cytokine IL-10, and Type III IFN stimulation product CXCL10/IP-10, we measured these parameters in INF individuals 6 months following anthelmintic treatment (post-T) and compared them to levels before treatment (pre-T). As shown in Figure 4B, INF individuals had significantly lower plasma levels of IL-10 (GM fold change of 1.08 compared to pre-T vs. post-T, *p* = 0.0144) and CXCL10/IP-10 (GM fold change of 1.20 compared to pre-T vs. post-T, *p* < 0.0001) at post-treatment in comparison to pre-treatment levels. Next, we wanted to determine, whether parasite antigen induces expression of CXCL10/IP-10 in *Ss* infection. We stimulated whole blood from INF and UN individuals with *Ss* antigen or PPD or LPS or media alone (unstimulated) and measured the level of CXL10/IP-10. As shown in Figure 4C, upon stimulation with parasite antigen, INF had significantly higher level of CXL10/IP-10 (GM of 261.2 pg/ml in INF compared to 211.5 pg/ml in UN, *p* = 0.0031). Conversely, there were no differences in the levels at baseline and those induced by PPD or LPS between the 2 groups. Thus, Ss infection is associated with increased systemic levels of IL-10 and CXCL10/IP-10 and increased levels of CXCL10/IP-10 when stimulated with parasite antigen.

### *Ss* infection is associated with increased numbers of DC subsets and reversal following treatment

**Figure 5:**
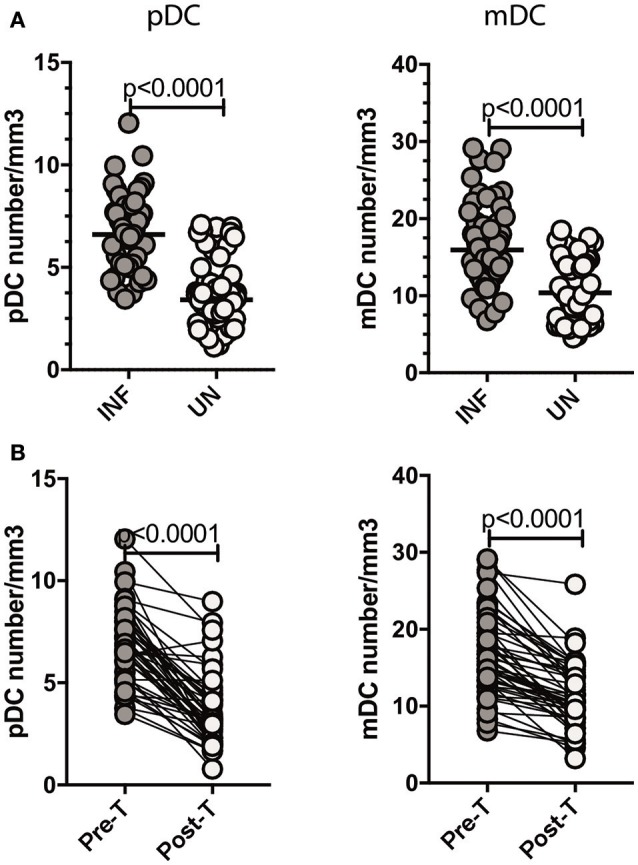
*Ss* infection is associated with increased numbers of pDC and mDC and reversal following treatment. **(A)** Absolute count of pDC and mDC in INF (*n* = 44) and UN (*n* = 44) individuals was measured by flow cytometry. The data are represented as scatter plots with each circle representing a single individual. *P*-values were calculated using the Mann–Whitney *U*-test with Holms correction for multiple comparisons. **(B)** Absolute count of pDC and mDC in INF (*n* = 44) individuals at pre-treatment [pre-T and 6 months following treatment [post-T]. Data are shown line diagrams with each line representing a single individual. *P*-values were calculated using the Wilcoxon matched pair test with Holms correction for multiple comparisons.

Since Type III IFNs are known to alter DC subset numbers and function, we sought to examine the levels of DC subsets in *Ss* infection (8, 22). To this end, we measured the absolute counts of pDC and mDC in INF and UN individuals. As shown in Figure 5A, INF had significantly increased numbers of both pDC (GM of 6.601/mm^3^ in INF compared to 3.414/mm^3^ in UN, *p* < 0.0001) and mDC (GM of 15.95/mm^3^ in INF compared to 10.37/mm^3^ in UN, *p* < 0.0001) when compared with UN individuals. To determine the effect of treatment on absolute numbers of DC subsets, we measured these parameters in INF individuals 6 months following anthelmintic treatment (post-T) and compared them to numbers before treatment (pre-T). As shown in Figure 5B, INF had significantly decreased numbers of pDC (GM fold change of 2.00 compared to pre-T vs. post-T, *p* < 0.0001) and mDC (GM fold change of 1.13, *p* < 0.0001) in comparison with post-treated levels. Therefore, *Ss* infection is associated with alterations in the subset distribution of DCs.

### pDC exhibit a positive relationship with IFN lambda-2 and IFN lambda-3 in *Ss* infection

**Figure 6:**
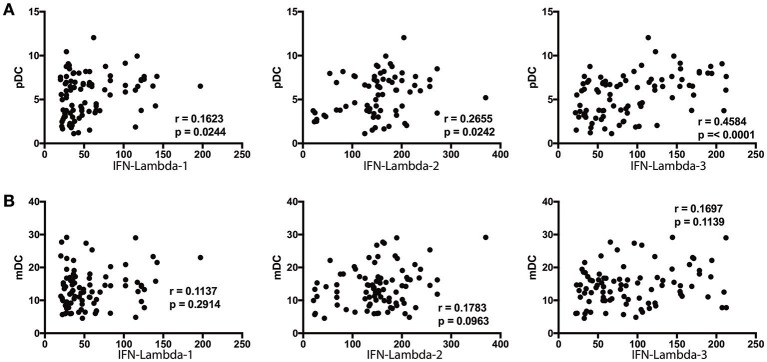
pDC exhibit a positive relationship with IFN-lambda-2 and IFN-lambda-3 in *Ss* infection. **(A)** The correlation between plasma levels of Type III IFNs (IFN-lambda-1, IFN-lambda-2, and IFN-lambda-3) and pDC in INF individuals are shown. **(B)** The correlation between plasma levels of Type III IFNs (IFN-lambda-1, IFN-lambda-2, and IFN-lambda-3) and mDC in INF individuals are shown. *P*-and *r*-values were calculated using the Spearman rank correlation test.

Since DC subsets and Type III IFNs are known to regulate each other, we sought to assess the relationship between the absolute numbers of pDC and mDC with systemic levels of Type III IFNs (8, 22). To this end, we performed Spearman rank correlation between these parameters. As shown in Figure 6A, the numbers of pDC exhibited a significant positive correlation with the levels of IFN lambda-2 (*r* = 0.2434; *p* = 0.0223) and IFN lambda-3 (*r* = 0.4584; *p* < 0.0001), whereas the numbers of mDC did not exhibit any significant correlation with the levels of IFN lambda-1, IFN lambda-2, or IFN lambda-3 (Figure 6B).

## Discussion

Type III IFNs were initially described as anti-viral agents that function at epithelial surfaces and that act similar to Type I IFNs (1, 2). Type III IFNs are produced by epithelial cells, pDC (predominantly), mDC, and monocytes (1, 2). Also, TLR and other pattern recognition receptor activation is a known mechanism for inducing Type III IFN production (1, 2). Type III IFNs are the dominant IFNs produced after viral infection in the respiratory tract and also plays a key role in gastrointestinal immunity (23, 24). Recent evidence, however, suggests that the influence of Type III IFNs on host immunity extends beyond its effect at the mucosal level to effects on systemic immune responses, specifically the innate, and adaptive arms of the immune response (25). Thus, Type III IFNs are known to shape adaptive immune responses to viral and bacterial infection, alter anti-tumor responses and affect immunity (1, 2, 26).

*Ss* infection is a soil transmitted helminth infection with the adult parasite residing in the small intestine and lasting for decades both because of its autoinfective lifecyle and because of its ability to modulate host immunity (13). The role of these (or other) helminth parasites in modulating innate responses such as the IFN response in not clear. To our knowledge, the present study is among the first to examine Type III IFNs in parasitic infection. Our study provides three important findings. First, our data show that *Ss* infection is associated with increased levels of IFN lambda-1, IFN lambda-2, and IFN lambda-3 in the circulation. This is clearly evident from the data contrasting systemic levels of Type III IFNs in INF and UN individuals. Our study also extends this observation to show that this association of elevated Type III IFNs is directly associated with helminth infection since anthelmintic treatment and presumed cure results in a significant reversal in the systemic levels of Type III IFNs with all three cytokines decreasing significantly. Our study also reveals that not all intestinal helminth infections induce this effect on Type III IFNs. Our examination of circulating as well as parasite—antigen stimulated levels of Type III IFNs clearly reveal no significant association of Type III IFN production with hookworm infections. One possible explanation for this discrepancy between intestinal helminths could be due to the fact that Ss infection has an auto-infective cycle with propensity for extra-intestinal migration.

Type III IFNs are part of the IL-10 superfamily of cytokines. The similarity in structure between IFN-λ and members of the IL-10 family probably reflects a common evolutionary origin as well as the evolutionary restraint caused by sharing a common receptor chain (3). An antagonistic role for IL-10 and type III IFNs has been suggested. Jordan et al. speculated that IL-10 may act as an antagonist to IL-29/IFN-λ1 functions, signifying a highly sensitive IL-10-dependent feedback mechanism that regulates the function of IL-29/IFN-λ1 and/or that IL-10 and IFN-λ compete for the IL-10R2/IL-10Rβ chain in their respective receptors. The IFN-λ receptor (IFNλR) is a heterodimeric receptor made up of IFNλR1 (also IL-28Rα) and IL-10Rβ subunits. Monocytes have been reported to produce IL-10 in response to IFN-λ (20). Inline with this earlier study, our study also demonstrate that INF individuals exhibited higher levels of IL-10. IFN-λ1 signaling also leads to the phosphorylation of STAT3, and Type-III interferon, IFN-λ1, can induce the expression of IP-10 (27). In agreement with earlier study, we have shown that the levels of CXCL10/IP-10 levels were increased in *Ss* infected individuals. Thus, Ss infection modulates and provokes Type III IFN expression and induces the production of IL-10 and CXCL10/IP-10.

Secondly, our study provides a mechanistic basis for the upregulation of Type III IFNs by demonstrating the induction of these cytokines by parasite antigens in INF individuals. The crude somatic antigens from *Ss* larvae presumably contain pathogen associated molecule patterns and therefore act as stimulants for the production of Type III IFNs. This is consistent with previous reports demonstrating the presence of a spectrum of helminth secreted proteins that act on host receptors including Toll like receptors or C-type lectin receptors (28). These include molecules such as ES-62 from *Acanthocheilonema vitiae*, that interacts with TLR4 (29) and excreted-secreted antigens from *Onchocerca volvulus*, that interacts with TLR2 (30). Interestingly, we did not observe any significant enhancement in the production of Type III IFNs upon non-parasite antigen (PPD) stimulation, indicating the infection specific nature of this immune response. Again, as with the systemic cytokines, our data also demonstrate a substantial reduction in the production of Type III IFNs from whole blood culture supernatants of INF individuals following treatment. This indicates and corroborates our first observation on the direct association between Type III IFN induction and *Ss* infection. At this stage, we do not have any evidence for the role of these cytokines in *Ss* infection. It is tempting to speculate, based on previous evidence that Type III IFNs help in maintaining the integrity of the epithelial barrier, that this barrier integrity is the main role for Type III IFNs in this intestinal helminth infection. In addition, Type 2 cytokines, including IL-4 are known inducers of Type III IFNs and in turn, Type III IFNs can modulate Type 1/Type 2 cytokine responses (7, 31, 32). Hence, it is possible that the predominant Type 2 environment in *Ss* infection drives the augmented Type III IFN response.

Thirdly, our study also reveals an alteration in the number of DC subsets in *Ss* infection. Thus, both pDC and mDC numbers are elevated in INF individuals and the elevation is reversed significantly post-treatment. Since, both pDC and mDC are known producers of Type III IFNs in viral infection (1, 2), we examined the association of Type III IFN with pDC and mDC. Our data clearly shown a positive relationship between pDC and IFN lambda-2 and IFN lambda-3, thus suggesting an association between the two parameters. Our study did not examine the exact source of Type III IFNs in *Ss* infection, although our whole blood culture results suggest that hematopoietic cells contribute to this production. Eosinophils were the only other cell type increased during infection but did not exhibit any significant correlation with Type III IFN levels. Future experiments will need to be performed to determine the source of these cytokines in helminth infections. However, our data on whole blood induced production of Type III IFNs in *Ss* infections and lack of heightenened plasma levels of Type III IFNs in hookworm infections suggest that hematopietic cells are major contributors of this response in this parasitic infection.

Our study, nevertheless, provides important insights into the regulation of Type III IFNs in *Ss* infection and its modulation post-therapy. Moreover, the role of Type III IFNs should be studied in gene-disrupted mice infected with helminth parasites to determine their effect on susceptibility, resistance, or pathology.

## Author contributions

SB conceived and designed the experiments. AR, SM, and YB performed the experiments. SB and AR analyzed the data. CD and TN contributed reagents, materials, analysis tools. TN and SB wrote the paper.

### Conflict of interest statement

The authors declare that the research was conducted in the absence of any commercial or financial relationships that could be construed as a potential conflict of interest.

## Supplementary material

Supplementary Figure 1 Gating strategy for DC subsets. A representative flow cytometry plot from an INF individual showing the gating strategy for estimation of plasmacytoid (pDC) and myeloid DCs (mDC). Plasmacytoid DC were classified as (Lin^−^ HLA-DR^+^ CD123^+^) and myeloid DCs as (Lin^−^ HLA-DR^+^ CD11c^+^).

Supplementary Figure 2 Hookworm infection is not associated with alterations in plasma, whole blood cell culture supernatants (unstimulated, parasite antigen, PPD, and LPS stimulated) levels of Type III IFNs. **(A)** The plasma levels of Type III IFNs, IFN-lambda-1, IFN-lambda-2, and IFN-lambda-3 were measured in hookworm-infected [INF] (*n* = 44) or un-infected [UN] (*n* = 44) individuals. The data are represented as scatter plots with each circle representing a single individual. *P*-values were calculated using the Mann–Whitney *U*-test with Holms correction for multiple comparisons. **(A)** The baseline or unstimulated (UNS) levels of Type III IFNs, IFN-lambda-1, IFN-lambda-2, and IFN-lambda-3 were measured in whole blood culture of hookworm -infected [INF] (*n* = 22) or un-infected [UN] (*n* = 22) individuals. **(B)** The parasite antigen (ES antigen) stimulated levels of Type III IFNs, IFN-lambda-1, IFN-lambda-2, and IFN-lambda-3 were measured in hookworm -infected [INF] (*n* = 22) or un-infected [UN] (*n* = 22) individuals. **(C)** The PPD stimulated levels of Type III IFNs, IL-28A, IL-28B, and IL-29. **(D)** The LPS stimulated levels of Type III IFNs, IL-28A, IL-28B, and IL-29 were measured in *Ss*-infected [INF] (*n* = 22) or un-infected [UN] (*n* = 22) individuals. Net cytokine levels are calculated by subtracting the antigen stimulated values from unstimulated values. The data are represented as scatter plots with each circle representing a single individual. *P*-values were calculated using the Mann–Whitney *U*-test with Holms correction for multiple comparisons.
