# Supplementary figures and images for: Elevated Systemic and Parasite—Antigen Stimulated Levels of Type III IFNs in a Chronic Helminth Infection and Reversal Following Anthelmintic Treatment
Source: Front Immunol. 2018 Oct 23;9:2353. doi: 10.3389/fimmu.2018.02353 (PMC6205947; doi:10.3389/fimmu.2018.02353)

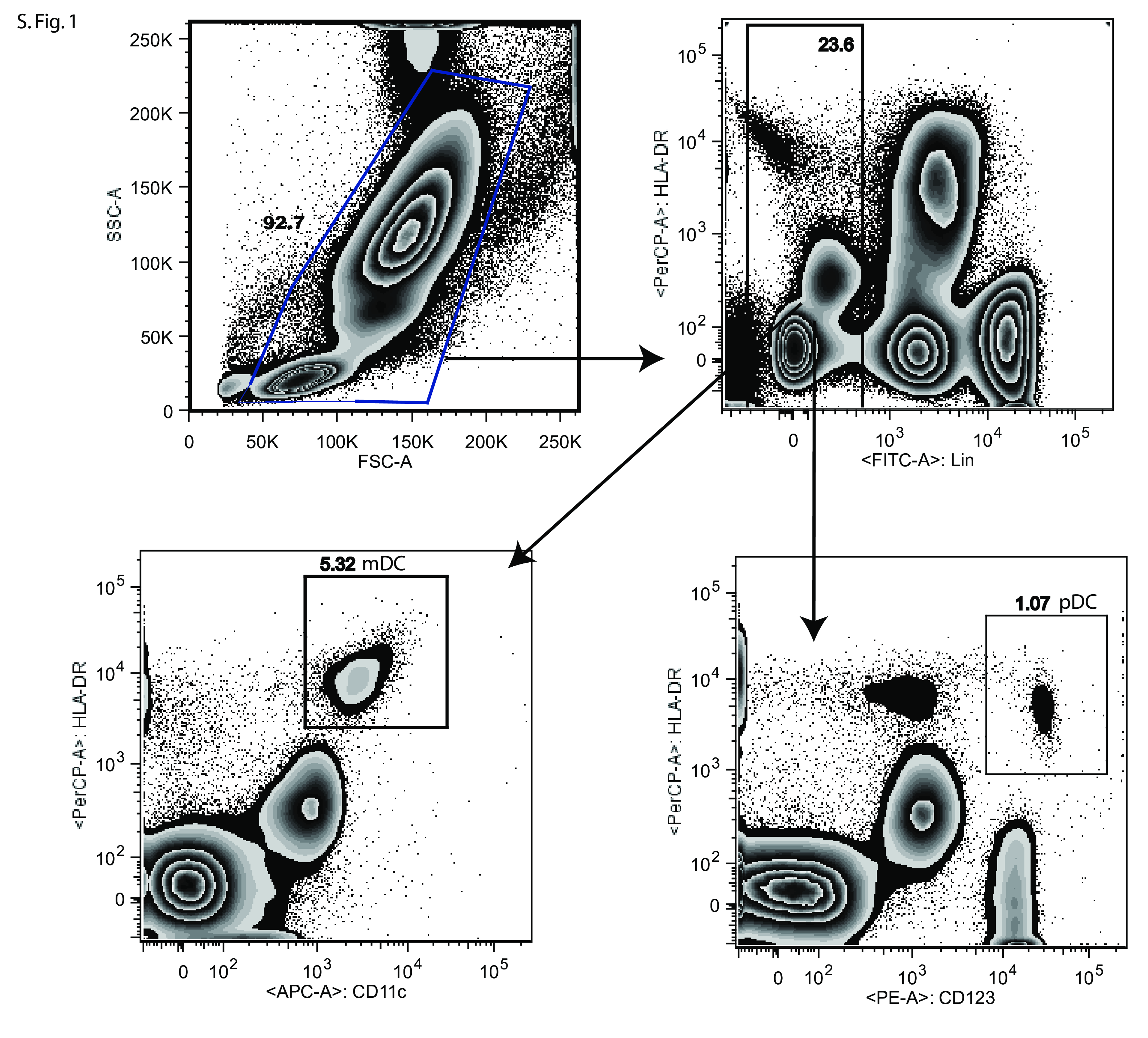

Supplement: Supplementary Figure 1 — Gating strategy for DC subsets. A representative flow cytometry plot from an INF individual showing the gating strategy for estimation of plasmacytoid (pDC) and myeloid DCs (mDC). Plasmacytoid DC were classified as (Lin− HLA-DR+ CD123+) and myeloid DCs as (Lin− HLA-DR+ CD11c+). [file Image_1.TIFF]

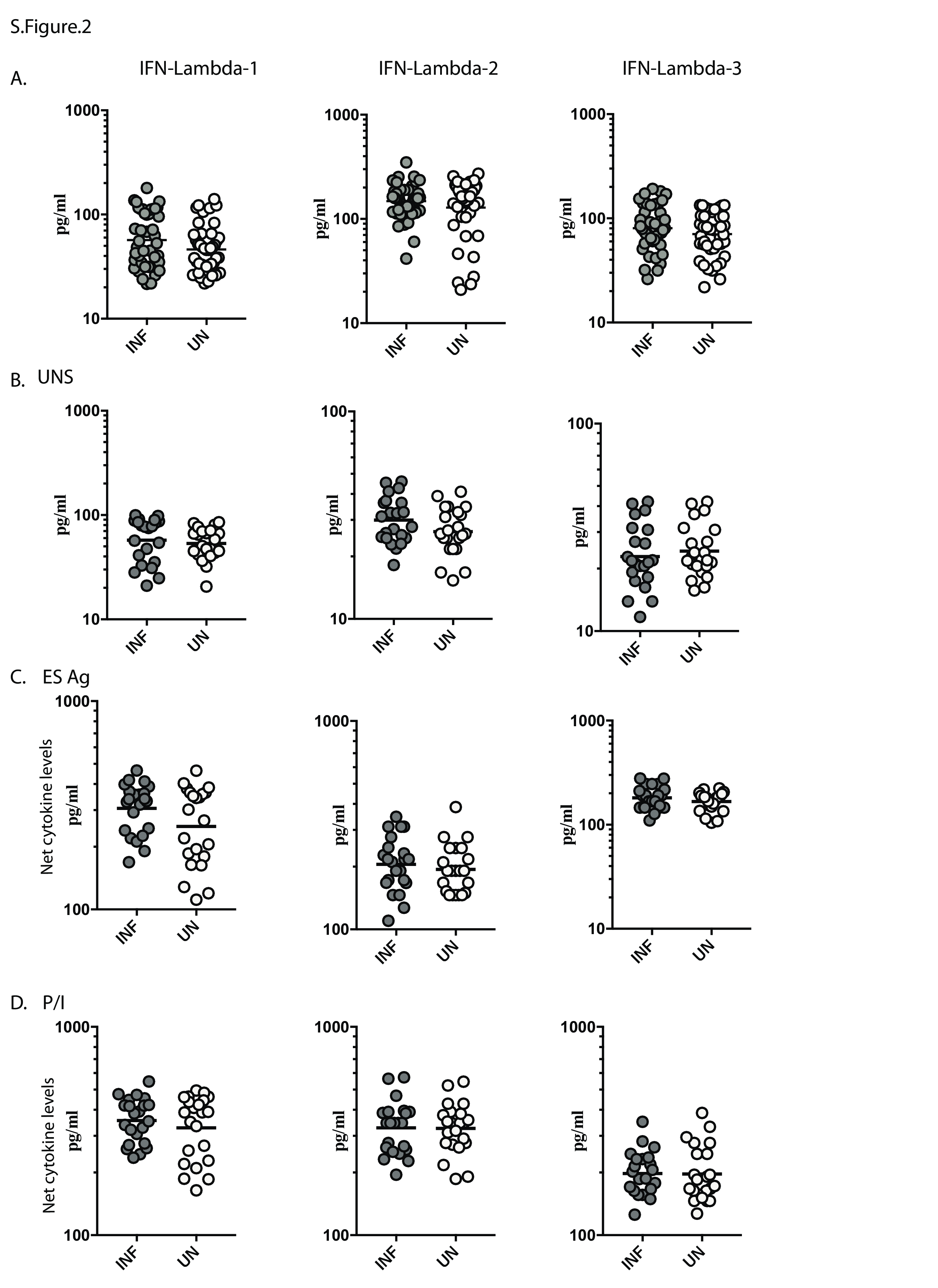

Supplement: Supplementary Figure 2 — Hookworm infection is not associated with alterations in plasma, whole blood cell culture supernatants (unstimulated, parasite antigen, PPD, and LPS stimulated) levels of Type III IFNs. (A) The plasma levels of Type III IFNs, IFN-lambda-1, IFN-lambda-2, and IFN-lambda-3 were measured in hookworm-infected [INF] (n = 44) or un-infected [UN] (n = 44) individuals. The data are represented as scatter plots with each circle representing a single individual. P-values were calculated using the Mann–Whitney U-test with Holms correction for multiple comparisons. (A) The baseline or unstimulated (UNS) levels of Type III IFNs, IFN-lambda-1, IFN-lambda-2, and IFN-lambda-3 were measured in whole blood culture of hookworm -infected [INF] (n = 22) or un-infected [UN] (n = 22) individuals. (B) The parasite antigen (ES antigen) stimulated levels of Type III IFNs, IFN-lambda-1, IFN-lambda-2, and IFN-lambda-3 were measured in hookworm -infected [INF] (n = 22) or un-infected [UN] (n = 22) individuals. (C) The PPD stimulated levels of Type III IFNs, IL-28A, IL-28B, and IL-29. (D) The LPS stimulated levels of Type III IFNs, IL-28A, IL-28B, and IL-29 were measured in Ss-infected [INF] (n = 22) or un-infected [UN] (n = 22) individuals. Net cytokine levels are calculated by subtracting the antigen stimulated values from unstimulated values. The data are represented as scatter plots with each circle representing a single individual. P-values were calculated using the Mann–Whitney U-test with Holms correction for multiple comparisons. [file Image_2.TIFF]
